# A Preliminary Assessment of the Role of Ambient Nitric Oxide Exposure in Hospitalization with Respiratory Syncytial Virus Bronchiolitis

**DOI:** 10.3390/ijerph13060578

**Published:** 2016-06-09

**Authors:** Nuredin I. Mohammed, Mark L. Everard, Jon G. Ayres, Nicola J. Barker, Ian J. Litchfield

**Affiliations:** 1 Institute of Applied Health Research, College of Medical and Dental Sciences, University of Birmingham, Birmingham B15 2TT, UK; nim007@bham.ac.uk (N.I.M.); j.g.ayres@bham.ac.uk (J.G.A.); 2 School of Paediatrics and Child Health, University of Western Australia, WA 6009, Australia; mark.everard@uwa.edu.au; 3 Sheffield Children’s NHS Foundation Trust, Respiratory Medicine, Sheffield S10 2TH, UK; nicki.barker@sch.nhs.uk

**Keywords:** respiratory syncytial virus, bronchiolitis, air pollution, nitrogen oxide

## Abstract

Some *in vitro* studies have indicated a possible link between respiratory syncytial virus (RSV) infection and exposure to Nitric Oxide (NO). However, these studies used much higher NO concentrations than normally found in the ambient environment. This preliminary study explored whether an association was present with short-term exposure to NO in the environment. RSV-related admission data between November 2011 and February 2012 were obtained from Sheffield Children’s Hospital. The dates of admission were linked to contemporaneous ambient NO derived from sentinel air monitors. The case-crossover design was used to study the relationship between daily RSV admissions and NO, controlling for temperature and relative humidity. We found little evidence of association between daily RSV admission rates and exposure to ambient NO at different lags or average exposure across several lags. The findings should, however, be viewed with caution due to the low number of events observed during the time frame. It is possible that the apparent lack of association may be accounted for by the timing of the seasonal RSV epidemic in relation to peaks in NO concentrations. A larger study incorporating a wider range of RSV and NO peaks would determine whether said peaks enhanced the number of RSV hospitalizations in children.

## 1. Introduction

Acute lower respiratory tract infections are the leading cause of child morbidity and mortality globally. Of the viral pathogens responsible, respiratory syncytial virus (RSV) is deemed one of the most significant [1,2]. The human respiratory syncytial virus is a paramyxovirus closely related to bovine and ovine RSV. It is believed to be transmitted via inhalation of droplets generated by coughing or self-inoculation into eyes and nose from contaminated hands. The virus only remains viable outside the human host for a short period [2,3]. Though the exact contribution of RSV is uncertain, it has been estimated that annually it is responsible for up to 200,000 deaths [4]. Figures from the UK indicate that RSV is the commonest cause of severe respiratory illness in young children aged under 2 years; it is also the most frequent cause of hospital admissions due to acute respiratory illness in young children, with over 8900 positive tests recorded by the Health Protection Agency (HPA) from October to March during 2012/2013 [5].

In temperate regions, annual epidemics of RSV-related infections typically follow a seasonal trend peaking during winter, while in tropical regions the epidemics are generally associated with the rainy season. A number of demographic factors have been associated with increased risk of RSV-related diseases including sex, age, birth during RSV season, siblings/crowding and previous RSV infection [6,7,8,9]. Due to the highly seasonal pattern of annual epidemics, meteorological factors such as temperature, hours of sunlight and humidity have been considered as potentially being related to RSV, but findings have been inconsistent, suggesting that such factors may not be affecting the incidence or severity of disease directly. Increased incidence of hospitalization has also been reported among infants living in industrialised areas compared to urban and rural areas [10,11] giving rise to the suggestion that there may be a link between certain air pollutants and severity of illness. Again, results from studies addressing this possibility have not been consistent [12,13,14,15].

One pollutant that has been little explored is nitric oxide (NO), which is known to be elevated during the winter compared to the summer season. These elevated levels of NO in winter may be due to reduced mixing of the lower air boundary during the winter months, and are further enhanced by reduced photochemical activity and behavioural changes due to the reduction in temperature [16]. A previous publication indicated that there may be a correlation between environmental levels of NO and admissions to hospital but this utilised aggregated data that were not subject to detailed analysis [17].

NO is of interest in the context of RSV infection for a number of reasons. *In vitro* work has shown that RSV infects macrophages and dendritic cells [18,19,20], two cells pivotal in orchestrating the immune response within the lungs and airways. Moreover, it has been shown that RSV may remain within the dendritic cell population in a latent form for prolonged periods. Reactivation of replication can be induced by exposing dendritic cells to NO at 600 ppb for two hours or by adding the NO donor S-nitroso-*N*-acetylpenicillamine (SNAP) [19,21]. RSV has been shown to up-regulate iNOS and nitrite production in a cell line affecting ion channel function and aspects of inflammation including up-regulating NF-kB [22]. In a rodent model of RSV infection in infants, administering an iNOS inhibitor to the young mice resulted in increased viral titres in bronchoalveolar lavage (BAL) samples though inflammation was reduced [23]. NO has also been shown to play important roles in host responses to the virus, affecting immune responses and apoptosis of host cells [24,25]. The present epidemiological study exploring the relationship between RSV and short-term exposure to ambient NO pollution was undertaken with the aim of exploring the possibility that environmental nitric oxide influences the severity of the clinical illness experienced by infants with RSV infection and hence the rates of hospitalization.

## 2. Materials and Methods 

### 2.1. Health Data

Data on daily admission of infants due to RSV infection were obtained from Sheffield Children’s Hospital (Sheffield Children’s NHS Foundation Trust) over a four month period (November 2011 to February 2012). Infection status was determined based on a positive test for RSV using a polymerase chain reaction (PCR) on nasopharyngeal aspirates. Few if any children with a simple upper respiratory tract infection are admitted to hospital and the study only addressed those admitted with a significant lower respiratory tract infection. All such children had a nasal sample taken for virus identification. In addition, date of birth, gender and date of admission (date of sampling) were provided for each child admitted to the hospital. Parents provided informed consent to participate in the study and ethical approval was obtained from Yorkshire and the Humber-Sheffield research ethics committee, National Research Ethics Service (NRES REC: 10/H1307/114).

### 2.2. NO Concentration Data

Corresponding data on ambient NO concentrations were drawn from three sentinel monitors managed and maintained by the Department for the Environment, Food and Rural Affairs (DEFRA) which provide hourly concentrations of NO for each 24 h period. Details on techniques of analysis, methods of sampling, precision and accuracy of measurements can be found from the DEFRA website [26]. One of the stations, Sheffield Centre, was an urban monitor located in the centre of Sheffield city. The second station, Sheffield Tinsley, was in a relatively industrial site about three miles north west of the city centre, while the third station, Ladybower, was located in a rural site some ten miles to the east of Sheffield city centre. Thus oxides of nitrogen sources in Sheffield are mainly from road traffic and industrial emissions. We presented descriptive statistics for all three sites but our statistical models were based on the daily average NO concentrations from the three monitors in Sheffield.

### 2.3. Climate Data

Data on daily minimum and maximum temperature for Sheffield were obtained from the Met office British Atmospheric Data Centre (BADC) [27]. The average daily temperature for the study was then calculated by taking the average of the minimum and maximum temperatures. In addition, data on daily levels of relative humidity were also obtained from the Met office.

### 2.4. Statistical Analysis

The case-crossover design was used to investigate the association between short-term exposure to NO and the occurrence of RSV admissions controlling for average daily temperature and relative humidity. This design, introduced by Maclure [28], has been widely applied in air pollution studies and is particularly useful for estimating the risk of a rare acute outcome associated with short-term exposure [29,30,31,32]. In case-crossover design, each case acts as their own control and like case-control studies [33] the distribution of exposure is compared between “cases” and “controls”. That is, exposure at the time just prior to the event (“case” or “index” time) is compared with a set of “control” times that represent the expected distribution of exposure for non-event follow-up times. The design helps primarily to control for confounding by subject-specific factors which do not change over time such as ethnicity and gender.

We applied the time stratified case-crossover approach where the strata are matching days based on the same day of the week, calendar month and year. That is, control days were selected from the same day of the week, within the same calendar month and year as the event day. Usually, analyses based on this design are carried out using a conditional logistic regression model. However, we applied a conditional Poisson regression model which has been shown to give estimates equivalent to the conditional logistic model [34]; the conditional Poisson model has the advantage of easily allowing for overdispersion and autocorrelation. All our models assume a linear effect of NO on RSV admissions while the effects of temperature and relative humidity are likely to be non-linear [35,36] and were modelled using natural cubic splines with three degrees of freedom. We explored various lag structures including single lags 0, 1, 2, …, 6 and corresponding average of lags 0–1, 0–2, 0–3, …, 0–6 for NO exposure. Additional sensitivity analysis was conducted using NO data from Sheffield Central monitoring station to compliment the primary analysis which was based on the average NO levels from the three stations. All analyses were performed using the R statistical package [37].

## 3. Results

**Figure 1:**
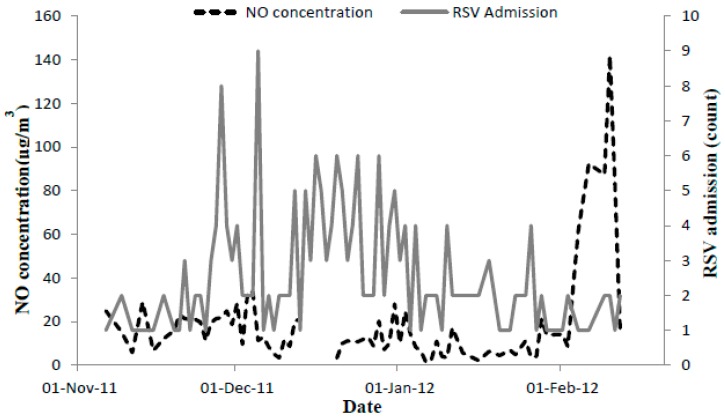
Average NO concentrations and RSV related admissions at Sheffield Children’s Hospital.

The average number of admissions per day was 1.7 and occurred in only 79 days of the study period, which spaned four months (Table 1). The mean age of a child admitted to the hospital was a little over 4 months (133 days) and all children were under the age of one year except one child who was a year and half old. Of the total admitted children, 99 (46.7%) were females and 109 (52.4%) were males. The peak admission counts were observed at the end of November and beginning of December as shown in Figure 1 (grey solid line) and did not seem to coincide with those peaks for the average NO concentrations which were observed in January and February.

**Figure 2:**
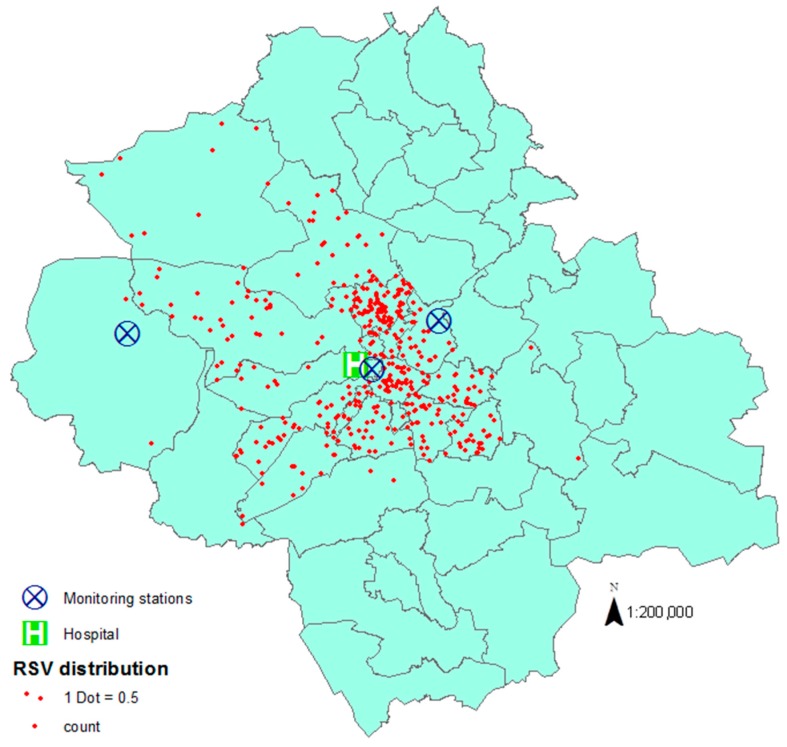
Distribution of RSV infected cases admitted to Sheffield Children’s Hospital and location of monitoring stations.

Most children admitted to Sheffield Children’s Hospital due to RSV infection were from locations surrounding central Sheffield, where the hospital itself as well as the central monitoring station are located (Figure 2).

**Figure 3:**
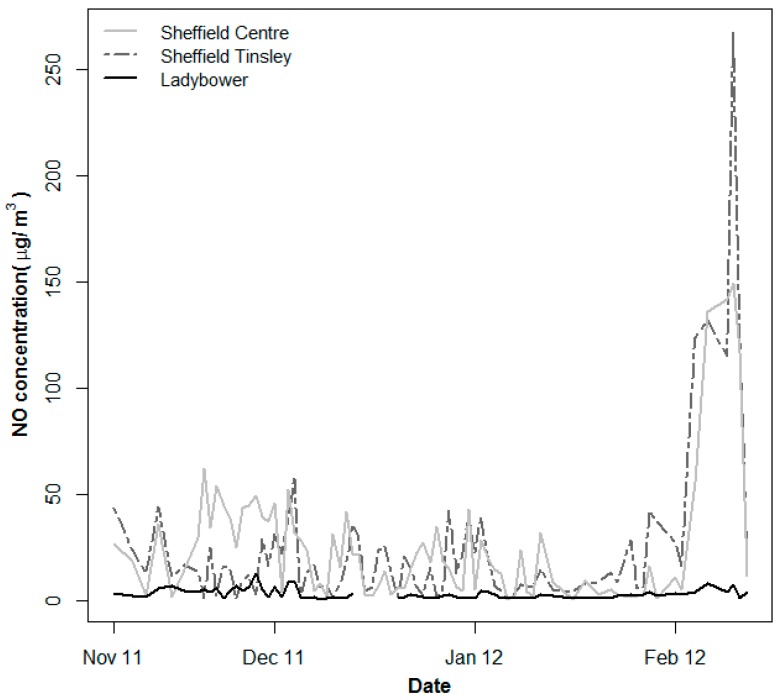
NO concentrations for the three monitoring stations around Sheffield city over the study period November 2011–February 2012.

As expected, NO concentrations were much lower at the rural Ladybower station while the urban Sheffield Centre and Sheffield Tinsley had more or less similar concentrations over the study period. For all the three stations, NO levels tend to show peaks in February (Figure 3). NO concentration data were missing for six days in Ladybower and one day for Sheffield Central stations. Relative humidity data were also missing for twenty days.

**Figure 4:**
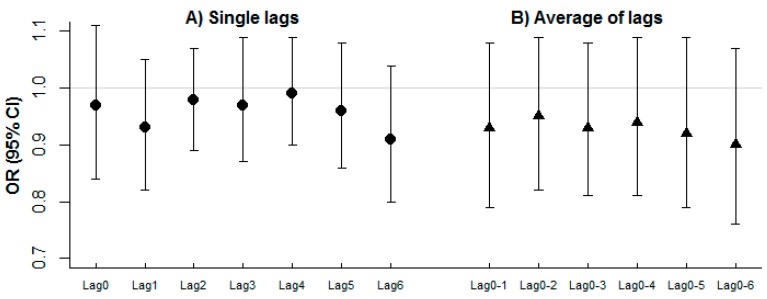
OR (95% CI) for the association of daily RSV admission and average NO concentrations in Sheffield. (**A**) Single lags; (**B**) Average of lags.

Overall, we did not find evidence of association between daily RSV admission rates and ambient NO concentrations in Sheffield; for example, the odds ratio (OR) (95% confidence interval (CI)) associated with a 10 μg/m^3^ increase in previous day (lag 1) NO concentration was 0.93 (0.82, 1.05) after controlling for non-linear effects of temperature and relative humidity. Results were similar across the various single lags and corresponding average of lags considered (Figure 4).

The results presented above were based on average NO exposure data from three stations, namely Sheffield Centre (urban site), Sheffield Tinsley (industrial site) and Ladybower (rural site) which had six missing observations. A sensitivity analysis using NO data from Sheffield Central monitoring station, which had only one missing observation, provided qualitatively similar results with no evidence of association and did not affect the overall conclusions (Table 2).

**Figure 5:**
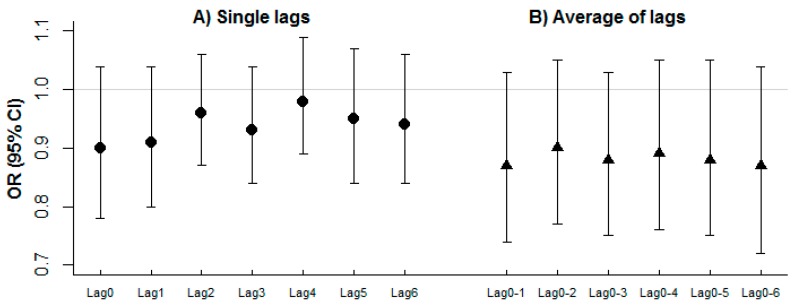
OR (95% CI) for the association of daily RSV admission and average NO concentrations without controlling for relative humidity. (**A**) Single lags; (**B**) Average of lags.

A further sensitivity analysis without adjusting for relative humidity was conducted in order to check whether the relatively higher missing rate (about 17%) in the humidity data affected the results. However, odds ratio estimates from this analysis were more or less similar to those based on models adjusting for relative humidity (Figure 5).

## 4. Discussion

The data generated in this study do not support the suggestion that environmental NO levels may influence the severity of RSV bronchiolitis, and hence hospitalizations rates, amongst infants infected during the annual epidemics. Our findings showed little association between ambient NO and RSV admissions. This was true for the various lags representing short-term exposure as well as analyses both with and without controlling for relative humidity. The latter analyses were conducted because while some studies showed significant association with RSV [7,8], others did not report such a relationship [12]. However, the results should be interpreted with caution due to limitations related to the data.

The daily number of events (RSV admissions) was very low in this data set—the reliability of our results could improve with more data in terms of both time and location. It is generally recommended to have a data set with an average of at least 10 event counts per day and large numbers of days in order to have reasonable power and precision for such environmental exposure studies [38]. Using a simulation-based approach we found the power could range from 62% to 92%, depending on the specific lag investigated, to detect a β coefficient (log odds) = 0.01. For example, in the case of the UK, one option would be to use the UK’s Royal College of General Practitioners weekly returns data for a national picture with a greater range of exposures and admissions rates. The case-crossover study design applied here to analyse the small data set is a reasonable method to deal with subject-specific confounding and has been successfully used in other epidemiological studies of rare disease outcomes. We found a negative result at the relatively lower NO concentrations that infants in this study were exposed to in comparison to the concentrations in the *in vitro* studies [19,21]. These studies used NO concentrations of 600 ppb, which is much higher than typical ambient levels [39].

The combustion of tobacco produces a range of chemicals including NO, and previous studies reported that both antenatal and postnatal exposure to tobacco within the home increases the incidence of hospitalization with RSV [40,41,42,43,44]. However, we believe such exposure to smoking is unlikely to be a confounder in our case-cross over study design, as little temporal variability is expected with respect to smoking status. That is, on average, the smoking behavior of households would not vary much with time.

There may, however, be another contributing factor to the apparent lack of an association observed here; the relative timing of the RSV epidemic and the peak in NO levels. It may be that NO cannot have an effect on RSV pathogenesis unless the two co-exist within a certain time period, say two or three days. If the RSV peak in a given year/area is not associated with particularly high NO then we might not see an effect. If the higher NO levels correspond with the RSV epidemic, this might then be associated with a greater number of cases; *i.e.*, NO acts as a potentiator of severity, as reflected in an increased number of attendances for medical help.

## 5. Conclusions

In summary, while we did not find an association between short-term exposure to ambient NO and RSV admissions, this preliminary study either did not have sufficient power to establish a potentially causal association or the lack of co-incidence of NO and RSV peaks meant that a potentiation of RSV infections was not able to be shown in this dataset.

## Figures and Tables

**Table 1:** Summary for daily admission counts and NO concentration by monitoring station.

Variable	Mean	Median (IQR ^b^)	Minimum	Maximum	Number of Days
Age (days)	132.7 (101)	111 (43–189)	7	573	121
Admission count	1.7 (1.9)	1 (0–2)	0	9	121
Temperature (°C)	6.3 (3.6)	6.7 (3.6–9.1)	−2.3	13.3	121
Relative humidity (%)	80.3 (11.4)	81.8 (72–88.5)	41.8	98	101
NO (μg/m^3^)					
Sheffield Centre	26.4 (31.7)	16.6 (5.1–36.8)	1	173.2	120
Sheffield Tinsley	25.8 (35.1)	15.7 (6.9–28.4)	1.3	267.9	121
Ladybower	3.2 (2.1)	2.5 (1.6–4.2)	0.6	12.9	115
Average	18.6 (20.7)	11.8 (7.2–21.1)	1.3	141.8	114
Sex (N, %) ^a^					
Female	99 (47.6)				

^a^ Number, percent; ^b^ Interquartile range.

**Table 2:** Association between NO and RSV admissions using data from a central monitoring station *vs*. average NO concentration from three monitoring stations.

Metric	Sheffield Centre		Average *	
	OR (95% CI)	*p*-Value	OR (95% CI)	*p*-Value
Lag 0	0.94 (0.85, 1.04)	0.23	0.97 (0.84, 1.11)	0.62
Lag 1	0.98 (0.91, 1.05)	0.57	0.93 (0.82, 1.05)	0.23
Lag 2	0.99 (0.92, 1.05)	0.69	0.98 (0.89, 1.07)	0.59
Lag 3	1.01 (0.95, 1.07)	0.80	0.97 (0.87, 1.09)	0.62
Lag 4	0.99 (0.93, 1.06)	0.80	0.99 (0.9, 1.09)	0.85
Lag 5	1.0 (0.93, 1.08)	0.97	0.96 (0.86, 1.08)	0.51
Lag 6	0.96 (0.88, 1.04)	0.27	0.91 (0.8, 1.04)	0.16
Lag 0–1	0.94 (0.85, 1.04)	0.27	0.93 (0.79, 1.08)	0.33
Lag 0–2	0.95 (0.87, 1.05)	0.34	0.95 (0.82, 1.09)	0.45
Lag 0–3	0.96 (0.87, 1.06)	0.40	0.93 (0.81, 1.08)	0.36
Lag 0–4	0.96 (0.86, 1.06)	0.39	0.94 (0.81, 1.09)	0.39
Lag 0–5	0.96 (0.85, 1.07)	0.43	0.92 (0.79, 1.09)	0.34
Lag 0–6	0.93 (0.82, 1.05)	0.24	0.9 (0.76, 1.07)	0.24

* The stations are Sheffield Centre (urban), Sheffield Tinsley (industrial) and Ladybower (rural).

## Abbreviations

The following abbreviations are used in this manuscript:

RSV	Respiratory syncytial virus
NO	Nitrogen Oxide
OR	Odds ratio
